# Efficacy of hyaluronic acid in temporomandibular disorders evaluated with diagnostic criteria for temporomandibular disorders (DC/TMD)

**DOI:** 10.1111/joor.13840

**Published:** 2024-08-29

**Authors:** Pankaj Kukreja, Bhavna Jha Kukreja, Maria Maddalena Marrapodi, Vincenzo Ronsivalle, Marco Cicciù, Giuseppe Minervini

**Affiliations:** ^1^ Department of Biomedical and Dental Sciences, Faculty of Dentistry Al‐Baha University Al‐Baha Kingdom of Saudi Arabia; ^2^ Assistant Professor, Periodontology College of Dentistry Preventive Dental Sciences Ajman United Arab Emirates; ^3^ Department of Woman, Child and General and Specialist Surgery University of Campania “Luigi Vanvitelli” Naples Italy; ^4^ Department of General Surgery and Medical‐Surgical Specialties, School of Dentistry University of Catania Catania Italy; ^5^ Saveetha Dental College and Hospitals, Saveetha Institute of Medical and Technical Sciences (SIMATS) Saveetha University Chennai Tamil Nadu India; ^6^ Multidisciplinary Department of Medical‐Surgical and Dental Specialties University of Campania Luigi Vanvitelli Caserta Italy

**Keywords:** corticosteroids, crepitus, DC/TMD, hyaluronic acid, temporomandibular disorders

## Abstract

**Background:**

The study aimed to retrospectively assess the efficacy of hyaluronic acid (HA) in managing temporomandibular disorders (TMD) using the diagnostic criteria for temporomandibular disorders (DC/TMD). There has been an ongoing debate regarding the effectiveness of HA as a treatment option for TMD, which necessitated a thorough evaluation.

**Methods:**

The review adhered to PRISMA guidelines conducted across eight different databases, including PubMed, Embase, Web of Science, Cochrane Library, Scopus, ScienceDirect, PsycINFO and CINAHL. The selection criteria included studies that evaluated the efficacy of HA in TMD patients, utilised DC/TMD, and were published in English. Data extraction and quality assessment were performed independently by two reviewers. ROB–2 tool was employed to assess methodological quality of the assessed studies.

**Results:**

A total of 10 studies met the inclusion criteria. They demonstrated that HA was effective in improving various symptoms of TMD, such as pain, mouth opening and joint sounds over control group. But on the other end, platelet‐rich plasma (PRP) was found to be better than HA intervention in alleviation of TMD symptoms. However, the degree of improvement varied across the studies. Some studies reported adverse effects, but these were typically minor and transient. Risk of bias assessment was low in all the included studies.

**Conclusion:**

The findings suggest that HA can be an effective treatment for TMD when evaluated with DC/TMD. However, the variation in effectiveness across studies indicates the need for individualised treatment planning and careful monitoring of adverse effects. Further research is needed to refine the treatment protocols and understand the long‐term effectiveness and safety of HA in TMD management.

## Graphical abstract

Hyaluronic acid (HA) was effective in improving various symptoms of temporomandibular disorders (TMD), such as pain, mouth opening and joint sounds over control group. But on the other end, platelet‐rich plasma (PRP) was found to be better than HA intervention in alleviation of TMD symptoms. However, the degree of improvement varied across the studies. Some studies reported adverse effects, but these were typically minor and transient.

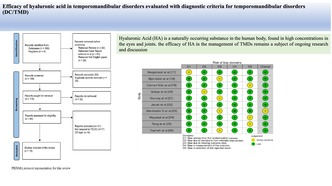

## 1 INTRODUCTION

When we talk about oral disorders or anomalies associated with the oral cavity, we generally tend to focus on oral lesions, different types of malignancies of the oral cavity and other specific pathologies more often than not. However temporomandibular disorders (TMDs) have started taking precedence as somewhat of a major concern when referring to diseases that affect the morphology and overall structure of the oral cavity, especially in terms of their recency where an observed increase in the incidence of TMDs has been documented within the past couple of decades.^1^, ^2^, ^3^

TMDs normally tend to encompass a group of conditions characterised by pain and dysfunction in the jaw joint and the muscles that control jaw movement.^4^, ^5^ The complexity of these disorders can be attributed to a variety of factors, including genetic predispositions, arthritis and jaw injuries.^2^, ^3^, ^6^ One common type is myofascial pain syndrome. This disorder primarily affects the muscles controlling jaw function and can also extend to the neck and shoulder muscles, causing pain and discomfort.^7^, ^8^, ^9^, ^10^, ^11^, ^12^ Another form of TMD is internal derangement of the joint, which involves a displaced disc, a dislocated jaw or injury to the condyle.^13^ The condyle is the rounded edge of the jaw that interacts with the temporal bone of the skull, and any damage to this area can lead to significant dysfunction and discomfort. Degenerative joint disease is another type of TMD that refers to conditions such as osteoarthritis or rheumatoid arthritis that specifically affect the jaw joint.^14^, ^15^, ^16^, ^17^, ^18^

Surgical options are often considered in treating TMDs, as patients generally believe that a surgical procedure might alleviate symptoms completely and address the root cause of the condition.^7^, ^8^, ^9^ However, it is vital to consider the complications and risks associated with such a modality, not to mention the overall success rate. When proceeding with a surgical approach, it is important to remember that patients are often unaware of these risks. Therefore, different types of procedures which tend to be less invasive and comparatively safer in terms of the things that can go wrong must also be looked at when we talk about management modalities associated with management of TMDs.^2^, ^4^, ^5^

Hyaluronic acid (HA) is a naturally occurring substance in the human body, found in high concentrations in the eyes and joints.^14^ It is a clear, gooey substance that is produced by the body to retain water and keep tissues well lubricated. In medicine, HA is used in a variety of treatments.^19^ It is often injected into the joints of individuals suffering from osteoarthritis, a degenerative joint disease, to help reduce pain and increase mobility. In ophthalmology, during certain eye surgeries including cataract removal, corneal transplantation, and repair of a detached retina, a small amount of HA is used to replace natural fluids. HA is used in TMD treatment due to its lubricating qualities, which can help improve the smooth movement of the jaw joint.^19^ It acts as a shock absorber in the joint and promotes the healing of the inflamed synovial membrane and cartilage of the joint. Injections of HA in the temporomandibular joint (TMJ) can help reduce pain and increase jaw mobility. Studies have shown that HA injections can be beneficial for patients with TMDs.^19^, ^20^, ^21^, ^22^, ^23^, ^24^, ^25^ They have been found to improve mouth opening, reduce joint noises, and decrease pain. The benefits of HA injections can last for several months and can be repeated if necessary^26^, ^27^, ^28^.

However, the efficacy of HA in the management of TMDs remains a subject of ongoing research and discussion. This review aims to evaluate the existing evidence on the efficacy of HA in treating TMDs, with a specific focus on studies that have utilised the DC/TMD. By honing in on research that uses these standardised and internationally recognised diagnostic criteria, we hope to provide a comprehensive and reliable overview of the role of HA in TMD management.

## 2 MATERIALS AND METHODS

### 2.1 Review design

**FIGURE 1:**
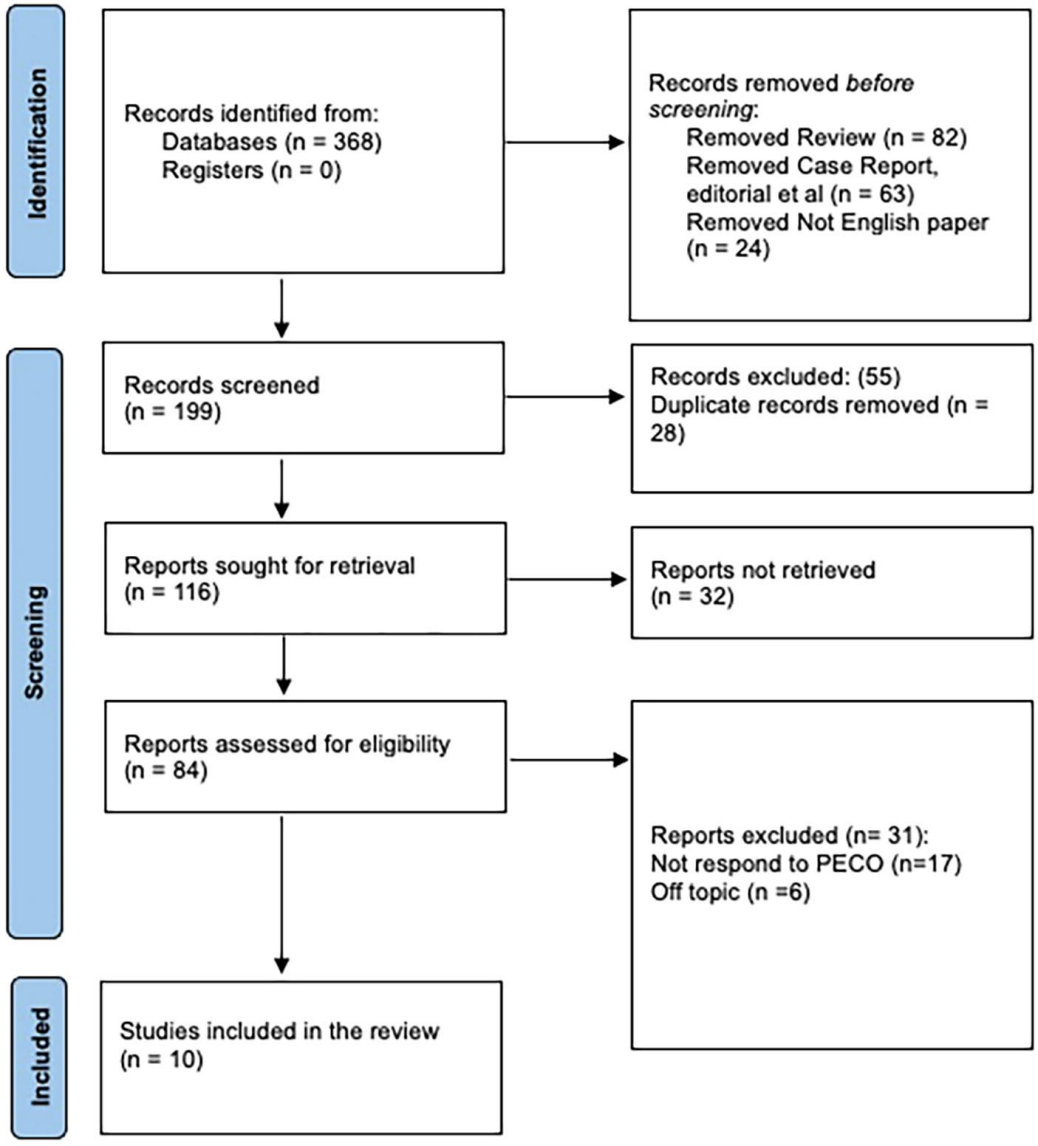
PRISMA protocol representation for this review.

This systematic review was conducted following the Preferred Reporting Items for Systematic Reviews and Meta‐Analyses (PRISMA) protocol.^29^ Figure 1 shows the different steps that were taken for selecting relevant clinical studies that were in accordance with the objectives delineated for this review (Table 1).

**TABLE 1:** Abbreviations used in the review.

Abbreviation	Full form
HA	Hyaluronic acid
PRP	Platelet‐rich plasma
ATS	Arthrocentesis
RL	Ringer's lactate
CS	Corticosteroids
SS	Saline solution
kDa	KiloDalton

The PECO (Population, Exposure, Comparator, Outcome) protocol for this review was defined as follows:

- Population (P): Adults diagnosed with TMD, as defined by DC/TMD, without any restrictions on demographic characteristics or severity of TMD.
- Exposure (E): Treatment with HA, irrespective of the dosage, formulation, frequency or duration of treatment.
- Comparator (C): The comparators included no treatment, placebo or other standard treatments for TMD.
- Outcome (O): Primary outcomes were improvements in TMD symptoms, including pain, mouth opening and joint sounds. Secondary outcomes included adverse effects reported with the use of HA.

### 2.2 Database search protocol

The systematic search for this review was conducted across eight databases: PubMed, Embase, Web of Science, Cochrane Library, Scopus, ScienceDirect, PsycINFO and CINAHL. We developed a comprehensive search strategy using Medical Subject Headings (MeSH) keywords, free‐text terms and Boolean operators to ensure the identification of all relevant studies as shown in Table 2.

**TABLE 2:** Search strings utilised across the databases.

Database	Search string
PubMed	(“hyaluronic acid”[MeSH Terms] OR “hyaluronic acid”[All Fields]) AND (“temporomandibular disorders”[MeSH Terms] OR “temporomandibular disorders”[All Fields]) AND (“diagnostic criteria for temporomandibular disorders”[MeSH Terms] OR DC/TMD[All Fields])
Embase	(‘hyaluronic acid’/exp OR ‘hyaluronic acid’) AND (‘temporomandibular disorders’/exp OR ‘temporomandibular disorders’) AND (‘diagnostic criteria for temporomandibular disorders’/exp OR ‘DC/TMD’)
Web of Science	TS = ((“hyaluronic acid” AND “temporomandibular disorders” AND “diagnostic criteria for temporomandibular disorders”) OR DC/TMD)
Cochrane Library	(“hyaluronic acid” in Title Abstract Keyword OR “hyaluronic acid” in MeSH Terms) AND (“temporomandibular disorders” in Title Abstract Keyword OR “temporomandibular disorders” in MeSH Terms) AND (“diagnostic criteria for temporomandibular disorders” in Title Abstract Keyword OR DC/TMD in MeSH Terms)
Scopus	(TITLE‐ABS‐KEY(“hyaluronic acid”) AND TITLE‐ABS‐KEY(“temporomandibular disorders”) AND TITLE‐ABS‐KEY(“diagnostic criteria for temporomandibular disorders”) OR TITLE‐ABS‐KEY(DC/TMD))
ScienceDirect	(“hyaluronic acid” AND “temporomandibular disorders” AND “diagnostic criteria for temporomandibular disorders” OR “DC/TMD”) in Abstract, Title, Keywords
PsycINFO	(AB(“hyaluronic acid”) OR TI(“hyaluronic acid”)) AND (AB(“temporomandibular disorders”) OR TI(“temporomandibular disorders”)) AND (AB(“diagnostic criteria for temporomandibular disorders”) OR TI(“DC/TMD”))
CINAHL	(MH “Hyaluronic Acid”) AND (MH “Temporomandibular Joint Disorders”) AND (MH “Diagnosis”) OR AB(DC/TMD)

### 2.3 Selection criteria

Table 3 shows the inclusion and exclusion criteria delineated for this review so as to ensure a precise and relevant selection of studies.

**TABLE 3:** Inclusion and exclusion criteria utilised in the review.

Criteria	Description
Inclusion	1. Studies involving adults diagnosed with temporomandibular disorders (TMD) as per the diagnostic criteria for temporomandibular disorders (DC/TMD) 2. Studies that evaluated the efficacy of hyaluronic acid for the management of TMD 3. Randomised controlled trials (RCTs), non‐randomised controlled trials, cohort studies, case–control studies and cross‐sectional studies 4. Studies that compared hyaluronic acid to no treatment, placebo or other standard treatments for TMD 5. Studies that reported on the primary outcomes including improvements in TMD symptoms such as pain, mouth opening and joint sounds, and secondary outcomes including adverse effects related to hyaluronic acid treatment
Exclusion	1. Studies involving patients with TMD due to trauma, malignancy, systemic diseases, or congenital disorders 2. Studies that did not use DC/TMD for diagnosing TMD 3. Case reports, review articles, editorials, letters to the editor and animal studies 4. Studies that did not include a comparator 5. Studies that did not report on the predefined primary and secondary outcomes 6. Studies with incomplete data or those for which full‐text articles were not available

### 2.4 Data extraction process

The data extraction process was carried out independently by two reviewers using a pre‐defined data extraction form. This form comprised sections for capturing information such as the study's authors, year of publication, country, study design, participant details, intervention (dosage, frequency and duration of HA treatment), comparator details, and outcomes (improvement in TMD symptoms and adverse effects). For each included study, the two reviewers independently extracted data. Discrepancies between the reviewers were resolved through discussion or, when necessary, with the mediation of a third reviewer. Once the data were extracted, a cross‐check was performed to ensure the accuracy and completeness of the data.

### 2.5 Bias assessment protocol

**FIGURE 2:**
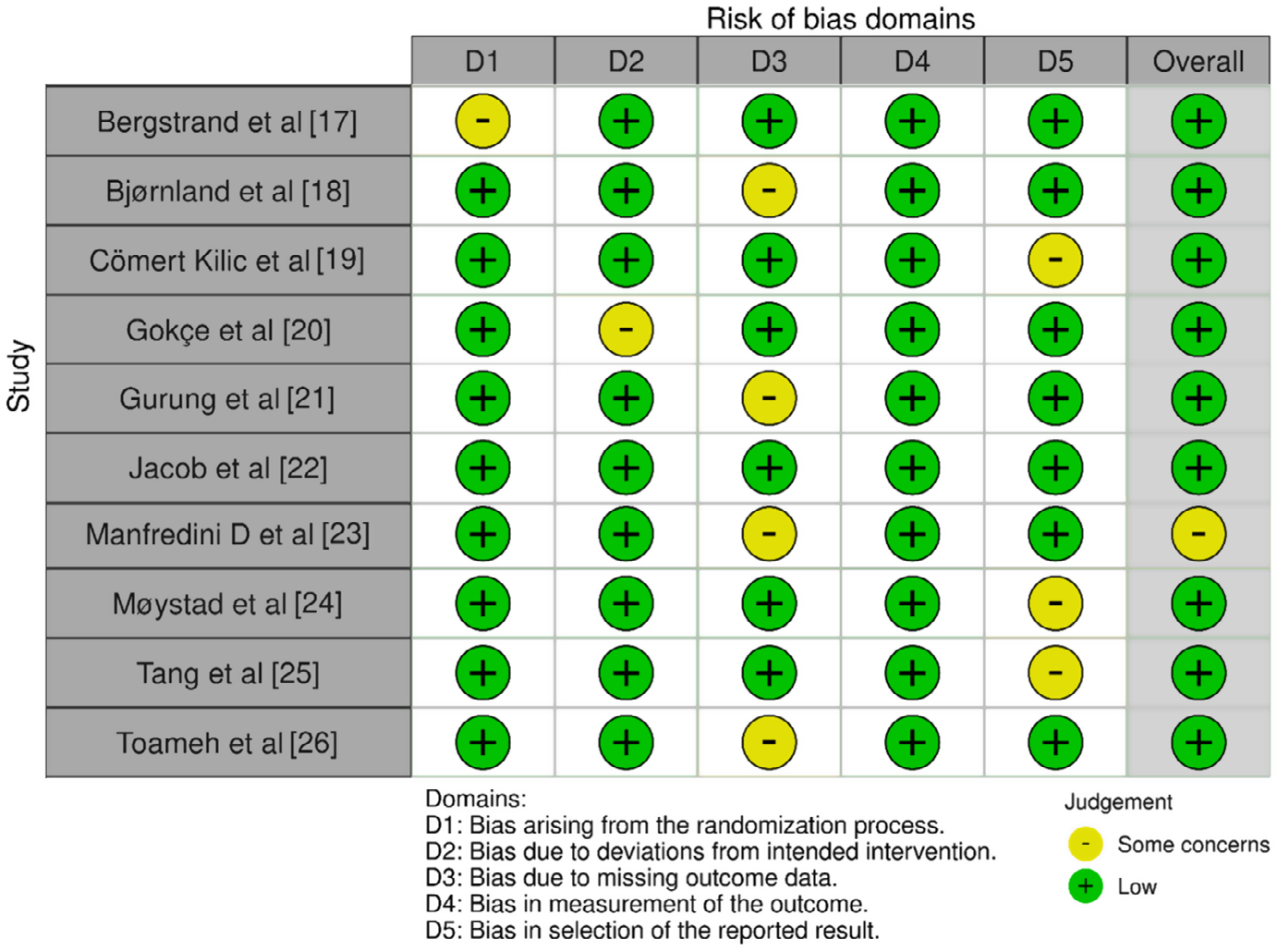
Assessed bias across different domains in the trials included in the review.

The protocol for assessing the risk of bias in the included studies was executed using the Cochrane Risk of Bias tool 2.0 (RoB 2.0).^30^ This tool was selected due to its comprehensive and detailed criteria for the evaluation of methodological quality in randomised trials. Two independent reviewers conducted the RoB assessment, any disagreements were resolved through discussion, or if necessary, a third reviewer was consulted. The results of this assessment have been elucidated through Figure 2.

## 3 RESULTS

### 3.1 Article selection

Initial identification of records yielded 368 potential studies from various databases, while no additional records were identified from registries. Prior to the screening phase, a total of 169 records were removed due to various reasons: 82 were reviews, 63 were either case reports or editorials and 24 were articles not published in English. This resulted in 199 records being advanced to the screening phase. During this phase, a further 55 records were excluded, leaving 144 records. Of these, 28 were identified as duplicates and were subsequently removed from the pool. The remaining 116 records were sought for retrieval. However, 32 reports could not be retrieved, reducing the number of records to be assessed for eligibility to 84. Subsequent assessment for eligibility resulted in the exclusion of 27 more reports. Among these, 16 reports did not respond to the PECO structure, while 6 were determined to be off‐topic. Following these exclusions, a total of 10 studies^31^, ^32^, ^33^, ^34^, ^35^, ^36^, ^37^, ^38^, ^39^, ^40^ were deemed eligible and were included in the final review.

### 3.2 Demographic characteristics

As evident through Table 4, a range of demographic variables were assessed across the included studies.^31^, ^32^, ^33^, ^34^, ^35^, ^36^, ^37^, ^38^, ^39^, ^40^ The studies were conducted between 2007 and 2023 and spanned several countries, including Norway,^31^, ^32^, ^38^ Turkey,^33^, ^34^ Italy,^37^ India,^35^, ^36^ China^39^ and Syria.^40^ The total sample size of the studies varied considerably, with the smallest sample consisting of 20 participants^35^ and the largest comprising 80 participants.^38^ The mean ages of the participants across studies also showed a wide range, from >18 years^35^ to 51.5 *±* 12.9 years.^38^ In terms of gender distribution, the majority of the participants were females in all studies.

**TABLE 4:** Demographic variables pertaining to the selected studies.

Study	Year	Region assessed	Total sample size (*n*)	Mean age (in years)	Gender ratio
Bergstrand et al.^14^	2019	Norway	37	47 ± 15.7	30 females
Bjørnland et al.^15^	2007	Norway	40	53.4 ± 12.9	34 females
Cömert Kilic et al.^16^	2016	Turkey	31	32.22 ± 14.33	26 females
Gokçe et al.^17^	2019	Turkey	60	37.4 ± 9.9	21 females
Gurung et al.^18^	2017	India	20	>18	6 females
Jacob et al.^19^	2021	India	47	46.19 ± 14.72	30 females
Manfredini et al.^20^	2012	Italy	72	50.1	51 females
Møystad et al.^21^	2008	Norway	80	51.5 ± 12.9	31 females
Tang et al.^22^	2010	China	40	42.67	31 females
Toameh et al.^23^	2019	Syria	30	38.87 ± 6.40	24 females

### 3.3 Assessed variables

Table 4 shows the HA‐related assessments observed in the included papers.^31^, ^32^, ^33^, ^34^, ^35^, ^36^, ^37^, ^38^, ^39^, ^40^ Bergstrand et al.^31^ conducted an RCT to compare two groups: one receiving arthroscopy and radiofrequency (ATS with RL) and the other receiving ATS with RL plus 1 mL of HA. The follow‐up period averaged 47 months. The study by Bjørnland et al.^32^ was an RCT that examined the effect of two 0.7–1 mL injections of 6000 kDa HA, administered 14 days apart. The follow‐up period was unspecified.

Cömert Kilic et al.^33^ performed an RCT comparing the effects of platelet‐rich plasma (PRP) and HA treatments in two separate groups, with a follow‐up period of 12 months. Gokçe et al.^34^ carried out an RCT comparing three different interventions: PRP, HA, and corticosteroid (CS) injections, each given at 1‐month intervals, with a follow‐up period of 3 months. In the study by Gurung et al.,^35^ an RCT was conducted to compare the effects of ATS with RL alone and ATS with RL plus HA, with follow‐ups conducted at several time points over a 3‐month period.

Jacob et al.^36^ performed an RCT comparing PRP, HA injections, and arthrocentesis alone, with follow‐ups at 1‐, 3‐ and 6‐month intervals. In the study by Møystad et al.,^38^ an RCT was conducted to assess the effects of two injections of Betamethasone, with follow‐ups at 2 weeks, 1 month and 6 months. Tang et al.^39^ conducted an RCT comparing the effects of HA injections and saline solution (SS) injections, with follow‐ups carried out during each injection and 1 week after the last injection. The study by Toameh et al.^40^ was an RCT comparing three groups: arthrocentesis only, arthrocentesis plus HA, and arthrocentesis plus PRP, with follow‐ups at 1‐, 3‐, 6‐ and 9‐month intervals.

#### 3.3.1 Overall inference

The comparison between HA intervention and PRP was assessed in five studies, with two studies^34^, ^40^ indicating that PRP was more effective in alleviating TMD symptoms, while the remaining three studies^33^, ^36^, ^37^ found no significant difference. Additionally, the comparison of HA treatment with conventional/control approaches in five studies revealed that three studies^35^, ^38^, ^39^ demonstrated superior results of HA over the control group, while the other two studies^31^, ^32^ showed no discernible difference. In summary, while favourable outcomes were observed for HA over the control group, PRP demonstrated better results than HA in alleviating TMD symptoms.

#### 3.3.2 Risk of bias assessment

All the studies showed good methodological quality as seen in Figure 2.

## 4 DISCUSSION

TMD involve a complex interplay of biomechanical, neurobiological, inflammatory and psychosocial factors. Pathophysiology often includes joint dysfunction, masticatory muscle dysfunction, central sensitization and inflammation. Treatment modalities range from conservative approaches like physical therapy, medications and splints to more invasive interventions such as arthrocentesis, injections and surgery.^1^ Each modality aims to address specific aspects of TMD pathophysiology, targeting pain reduction, restoration of joint function and improvement of quality of life. Multidisciplinary approaches tailored to individual patient needs often yield the best outcomes in managing TMD.

Overall TMD interventions can be categorised into three sections: conservative treatment, invasive procedures, and complementary and alternate therapies. Conservative treatment includes physical therapy, medication and oral appliances. Physical therapy involves a variety of techniques, including exercises, manual therapy and modalities like ultrasound and heat/cold therapy. These methods aim to enhance jaw function, alleviate pain, and restore mobility in individuals with TMD. Medications, such as nonsteroidal anti‐inflammatory drugs (NSAIDs), muscle relaxants and analgesics, are commonly prescribed to alleviate pain and inflammation associated with TMD. Oral appliances, such as splints or mouthguards, are used to reposition the jaw, reduce teeth grinding (bruxism) and alleviate muscle tension, offering a non‐invasive approach to managing TMD symptoms.^2^

Invasive procedures include intra‐articular injections, arthrocentesis, and surgery. Intra‐articular injections involve the administration of CSs, HA, or PRP directly into the TMJ to reduce inflammation and pain. Arthrocentesis is a minimally invasive procedure that entails the irrigation and aspiration of the TMJ to remove inflammatory mediators and enhance joint function. Surgery may be considered in severe cases of TMD to address structural abnormalities or joint degeneration. Surgical interventions such as arthroscopy, arthroplasty, or joint replacement aim to alleviate pain and restore function.

Complementary and alternative therapies include acupuncture, chiropractic care and nutritional supplements. Acupuncture, a traditional Chinese medicine technique, involves the insertion of thin needles at specific points to alleviate pain and promote relaxation, offering an alternative approach to managing TMD symptoms. Chiropractic care focuses on spinal manipulation and adjustments to improve jaw alignment and reduce muscle tension, complementing conventional treatment approaches for TMD. Nutritional supplements, including glucosamine, chondroitin and omega‐3 fatty acids, are believed to support joint health and reduce inflammation, offering potential adjunctive therapies for individuals with TMD.

HA is a naturally occurring glycosaminoglycan present in the synovial fluid of the TMJ, where it plays a crucial role in lubricating and cushioning the joint surfaces, as well as in regulating inflammation and tissue homeostasis. Recent findings suggest that HA might interact with several biological pathways implicated in TMD pathophysiology to exert its therapeutic effects. HA can affect joint lubrication and shock absorption. HA's viscoelastic properties enable it to lubricate the TMJ surfaces and provide shock absorption during jaw movements, thereby reducing friction and wear on the joint structures. By improving joint biomechanics, HA supplementation may alleviate mechanical stress and mitigate the progression of TMD symptoms associated with joint dysfunction^3^

HA exhibits anti‐inflammatory properties by inhibiting pro‐inflammatory cytokines, such as interleukin‐1 beta (IL‐1β) and tumour necrosis factor‐alpha (TNF‐α), and modulating the activity of immune cells involved in joint inflammation. HA has been shown to promote tissue repair and regeneration by stimulating the synthesis of extracellular matrix (ECM) components, enhancing cellular proliferation and migration, and modulating fibroblast and chondrocyte activity. In TMD, where structural damage to the TMJ disc, cartilage and surrounding tissues may occur, HA supplementation could support tissue healing and regeneration, thereby improving joint function and reducing symptoms. Literature evidence suggests that HA may have neuroprotective effects by modulating neuronal excitability, neurotransmitter release, and synaptic transmission within the central nervous system. Given the involvement of neurobiological mechanisms, such as central sensitization and altered pain processing, in TMD pathophysiology, HA therapy may exert analgesic effects by modulating neuronal activity and pain signalling pathways^41^, ^42^, ^43^.

As evident by the findings, the included studies offer a comprehensive overview of various interventions and their impacts on conditions related to MIO, lateral movements, pain and joint sounds. Bergstrand et al.,^31^ while noting no significant difference between the two studied groups, did highlight a significant increase in maximum opening and decrease in pain for both groups. This suggests that while individual treatments might not have had significantly different outcomes, they were still effective in improving the condition of the patients. The study by Bjørnland et al.^32^ reported a decrease in crepitus in both groups, with a notable increase in maximum opening and protrusion specifically in the HA group. This pointed to potential benefits of HA treatment, despite the overall similar progress in both groups. Cömert Kilic et al.^33^ observed no statistically significant difference in changes of pain level or maximum opening between the two groups, suggesting that both interventions had similar efficacy. Importantly, no complications were reported, adding weight to the safety of these treatments. In Gokçe et al.^34^ study, statistically significant changes in pain reduction were observed with the use of PRP and CS. Particularly, PRP showed greater efficacy compared to HA and CS, providing a potential preference for PRP in pain management.

Studies by Gurung et al.^35^ and Jacob et al.^36^ showed significant pain reduction and improvement in movements in both groups, with superior improvements in the group with HA. However, Gurung et al.^18^ reported transient facial paralysis after anaesthesia in four patients, highlighting potential risks associated with anaesthesia. Manfredini et al.^37^ saw improvements in all groups, with the most significant improvement seen in Group E in the latter study. However, Group D in the study by Manfredini et al.^20^ had to be discontinued due to severe post‐injection pain, underscoring the need for careful management of treatment side effects. Møystad et al.^38^ found that injections provided more relief to patients with joint pain only, with pain reduction being more in the HA group than the CS group. This suggested that the type of pain may influence the effectiveness of the treatment. In Tang et al.^39^ study, PAS levels in synovial fluid samples were higher in osteoarthritis patients (Group A + control) than in healthy volunteers. Group A showed significant pain reduction and PAS concentration improvement, pointing to the potential efficacy of this treatment for osteoarthritis patients. Toameh et al.^40^ found that all groups had significant increases in maximum mouth opening (MMO) and masticatory efficiency, and decreases in pain intensity over the 9 months. The PRP group showed the most improvement, followed by the HA group, while the control group showed smaller improvements.

Certain other studies which used HA to treat TMDs were also found in literature. The study by Attia et al.^24^ was an RCT was conducted to compare the efficacy of HA and PRP injections with HA and CS injections. Follow‐up assessments were carried out 1 week, 1 month and 6 months post‐operatively. Both groups showed improvement in MIO and lateral movements, and a reduction in clicking sounds. Notably, the pain reduction differed in terms of timeframes in the HA + PRP group (after 6 months) and the HA + CS group (after 1 week), indicating that while both treatments were effective, their impact was felt at different times. The study by Guarda et al.^25^ was a non‐randomised controlled trial, which involved five sessions of ATS with RL and HA injections, with follow‐ups carried out at several intervals over a 1‐year period. All evaluated parameters, especially pain at rest and while chewing, chewing efficiency, and functional limitations, saw significant improvements. Adverse effects not mentioned Guarda‐Nardini et al.^29^ conducted an RCT comparing two groups receiving ATS sessions with different HA concentrations, with each session followed up over a 3‐month period. Both protocols effectively improved symptoms for up to 3 months. No significant differences between the groups. No adverse effects reported, though anaesthetic injection caused discomfort. Guarda‐Nardini et al.^30^ study saw the group using the highest dosage of HA + ATS showing significant improvements in terms of pain and overall effect of treatment, with no adverse effects reported. This indicated the potential superiority of the treatment protocol used of HA in conjunction with ATS. Manfredini et al.^31^ conducted a non‐randomised controlled trial involving five ATS sessions with RL and HA injections, with follow‐ups conducted at several intervals over a 6‐month period. Notable improvement over time in chewing efficiency, perceived treatment effectiveness, functional limitation and pain when chewing. Adverse effects were not mentioned.

In comparison to the reviews of Derwich et al.^32^ and Xu et al.^33^, our review also examines the effectiveness of HA as well as other compounds to a lesser extent such as CSs, and PRP in treating TMDs. However, there are some notable differences in the outcomes and interpretations of these studies. In the review by Derwich et al.,^32^ it was concluded that arthrocentesis alone effectively reduces pain and improves jaw function in patients diagnosed with TMJ OA. They found that additional injections of HA or CS at the end of the arthrocentesis do not improve the final clinical outcomes. On the contrary, our study found superior improvements in groups treated with HA, indicating that HA might have a beneficial role when combined with other treatments. Derwich et al.^32^ also raised concerns about the potential negative effects of CS on the articular cartilage, suggesting that the use of CS in TMJ OA treatment should not be recommended and further examined. This is in line with the findings of our study, where one group in the study by Manfredini et al.^37^ had to be discontinued due to severe post‐injection pain, underscoring the need for careful management of treatment side effects.

The study by Xu et al.^33^ found that PRP and platelet‐rich fibrin (PRF) exhibited similar short‐term efficacy in treating TMD, with PRF showing more long‐term benefits. This is somewhat in contrast with our findings, where PRP showed greater effectiveness in pain reduction compared to HA and CS, suggesting a potential preference for PRP in pain management. In terms of MMO, Xu et al.^33^ found the effect of PRP to be superior after 1 month, but PRF provided more promising results after 3 and 6 months. In our study, multiple trials reported significant increases in MMO across different treatment groups, with the PRP group often showing the most improvement.

Tran et al.^34^ discuss the prevalence of OA, emphasising the need for effective treatments, and address the use of NSAIDs, intra‐articular injection of corticosteroids (IA‐CS), and intra‐articular injection of hyaluronic acid (IA‐HA) as common nonoperative management approaches. They also mention the potential placebo effect in these treatments and the variable effectiveness of IA‐HA in different joints. This is somewhat in line with our study, where HA and CS showed significant effects in pain reduction and improvement in jaw function.

However, Tran et al.^34^ also highlight the effectiveness of IA saline injection, often used as a placebo, in providing substantial pain relief in OA. This topic was not specifically addressed in our study, suggesting a potential area of exploration for future research. Ferreira et al. focus on the biological mechanisms involved in TMD, with an emphasis on the role of HA in the ECM. They discuss the dual role of HA, with high molecular weight HA acting as an anti‐inflammatory and low molecular weight HA as a pro‐inflammatory marker. This provides a deeper understanding of the possible mechanisms behind the effectiveness of HA in treating TMDs observed in our study^44^, ^45^.

The exact pathways through which HA injections exert their influence in the realm of TMDs are yet to be conclusively established. Nevertheless, it is hypothesized that HA engages in various cellular communications and impacts diverse cell classes within the joint milieu, subsequently preserving the equilibrium of synovial articulations. As endogenous HA is integral to the signalling of tissue injury and the ensuing repair processes, it is conjectured that externally sourced HA would similarly be involved in these intricate mechanisms. Intra‐articular HA injections are believed to exhibit anti‐inflammatory and analgesic properties, in addition to safeguarding the tissues within the joint. This is thought to be achieved through the interactions of HA with multiple cellular receptors, including but not limited to the CD44 receptor, the intercellular adhesion molecule‐1 (ICAM‐1), and the hyaluronan‐mediated motility receptor (HMMR)^35^, ^36^, ^37^.

The anti‐inflammatory effects elicited by HA are primarily attributed to its binding affinity with the CD44 receptor. Certain scholarly accounts propose that HA aids in curbing the expression of IL‐1β and TNF‐α^26^, ^27^ This suppression of these predominant catabolic cytokines in TMDs is likely to lead to a global decrease in the manifestation of pro‐inflammatory molecules. A recent clinical investigation reported a significant diminution in the levels of IL‐1β and IL‐18 following HA injection in a patient with TMD. Tang et al.^22^, one of the studies included in our review, also observed a substantial drop in the primary constituents of the plasminogen activator system in the synovial fluid of TMD patients post intra‐articular HA injections. The reduction of IL‐1β, in turn, is believed to cause a subsequent decrease in the activation of matrix metalloproteinases (MMPs) 1, 2, 3, 9 and 13, leading to a slowdown in the degradation of the ECM within the articular cartilage and promoting chondrocyte apoptosis. The attenuation of inflammation is further bolstered by a decrease in the expression of other pro‐inflammatory mediators, such as IL‐6, IL‐8, Prostaglandin E2 and free radicals, coupled with an increase in anti‐inflammatory cytokines.

On comparing and complementing HA with existing interventions, HA injections present a minimally invasive approach to managing TMD‐related pain and inflammation, akin to CS injections but with the potential for longer‐lasting effects. HA's viscoelastic properties serve to lubricate and cushion the TMJ, complementing the mechanical benefits offered by oral appliances and physical therapy exercises. Furthermore, HA's anti‐inflammatory effects can synergize with NSAIDs and other medications, providing additional relief from pain and inflammation. Importantly, HA can be integrated into multimodal treatment approaches for TMD, working in tandem with other interventions such as physical therapy or oral appliances. This comprehensive approach addresses both symptomatic relief and underlying biomechanical issues associated with TMD, potentially enhancing treatment outcomes for affected individuals.

### 4.1 Recommendations pertaining to clinical practice

Based on the assessed findings, it can be recommended that HA appears to be an effective treatment for managing symptoms of TMD, such as pain, limitations in mouth opening and joint sounds. However, it is important to consider that the extent of improvement and the most effective intervention may vary between individuals. This suggests the need for personalised treatment planning based on the specific needs and responses of each patient. Furthermore, while some minor and transient adverse effects were associated with the use of HA, the safety profile was generally favourable. Consequently, healthcare providers should ensure careful monitoring for potential adverse effects during treatment. Additionally, patients should be counselled about these potential effects and reassured that they are typically minor and temporary. While HA shows promises as a treatment for TMD, further research is needed to refine treatment protocols, understand the long‐term effectiveness and safety of HA, and determine the best practices for its use in managing TMD. This research should aim to establish standardised treatment protocols and guidelines to ensure optimal patient outcomes. It is also worth noting the importance of using validated diagnostic criteria, such as the DC/TMD, in evaluating the effectiveness of TMD treatments. This ensures a high level of rigour and consistency in clinical practice and research, helping to produce reliable, comparable findings that can inform future treatment decisions.

### 4.2 Limitations

There were several limitations in our review that should be acknowledged. First, the included studies exhibited heterogeneity in terms of the patient populations, HA formulations, and treatment protocols used. This variability might have influenced the observed effects of HA, limiting our ability to draw firm conclusions about its efficacy in all TMD patients. Also, the follow‐up period varied among the studies, and some had relatively short follow‐up periods. This limited our ability to evaluate the long‐term efficacy and safety of HA. Additionally, patient adherence to treatment protocols was not consistently reported, which could affect the interpretation of the findings. Prior treatments for TMD, including medications, physical therapy, splints, or other interventions, could affect the response to HA treatment. Participants with a history of multiple treatments may respond differently than those receiving HA as a first‐line therapy.

## 5 CONCLUSION

The overall findings of our study suggest that HA can be an effective treatment option for managing TMDs (Table 5). The observed improvements spanned various symptoms of TMD, including pain relief, increased mouth opening, and reduction in joint sounds, indicating the broad potential benefits of HA. However, these findings should be interpreted in the context of individual variability in response to treatment. The degree of improvement and the most effective intervention showed variation across the studies, emphasising the need for personalised treatment planning. Moreover, while some minor and transient adverse effects were reported, the overall safety profile of HA was generally favourable, suggesting that the benefits of treatment potentially outweigh the risks. Furthermore, our study reinforces the importance of using validated diagnostic criteria, such as DC/TMD, in evaluating the effectiveness of TMD treatments. This approach ensures a high level of rigour and consistency in clinical practice and research, which is essential for producing reliable and comparable findings.

**TABLE 5:** Inferences pertaining to the impact of HA on different types of TMDs observed.

Study	Design	Intervention assessed	Follow‐up period	Key findings assessed
Bergstrand et al.^14^	RCT	Two groups: (A) ATS with RL, (B) ATS with RL + 1 mL of HA (6000 kDa)	Average 47 months (25–79 months)	No significant difference between both groups was noted for Incisor opening (*p* = .223), though Group A showed 5 mm increase and Gp B showed 6 mm increase. No significant changes in joint sounds (*p* = .084) between groups. ATS reduced TMJ pain in the long term, but the use of HA did not demonstrate significant changes
Bjørnland et al.^15^	RCT	Group HA: Two 0.7–1 mL injections of HA, 14 days apart Group C: Two injections of betametasone sodium phosphate betametasone acetate, 14 days apart	14 days, 1 and 6 months	Pain severity was lesser in HA group as compared to Group C (p‐ = .0001). Both groups observed decrease in crepitus. Notable increase in maximum opening and protrusion in both groups Patients with pain benefitted from HA injections
Cömert Kilic et al.^16^	RCT	Two groups: PRP group: initial ATS with RL together with injection of PRP and then four injections of PRP alone, HA group: ATS with RL together with a single injection of 2 mL HA (500–700 kDa)	12 months	No statistically significant difference in changes of pain level or maximum opening between the two groups. No complications reported. Clinical efficiency of both groups were similar
Gokçe et al.^17^	RCT	Three groups: (1) Infiltration of 1 mL of PRP with 1‐month intervals, (2) Infiltration of 1 mL of HA with 1‐month intervals, (3) Infiltration of 1 mL of CS (Triamcinolone) with 1‐month intervals	3 months	Statistically significant changes in pain reduction with the use of PRP and CS. Greater efficacy of PRP compared to HA and CS
Gurung et al.^18^	RCT	Two groups: (A) 5 ATS RL with 1 week intervals, (B) 5 sessions of ATS with RL + HA with 1 week intervals.	First day, fifth day, 1 week, 1, 1½ and 3 months.	Significant pain reduction and improvement in movements in both groups. Superior improvements in group with HA was noted. Pain reduced to 2.40 ± 1.01 in Group A while it was 1.30 ± 0.48 in Group B (*p* = .007).Movement in Group A measured 42.50 ± 2.36 mm as compared to Group B with 45.60 ± 1.83 mm (*p* = .004). Four patients had transitory facial paralysis after anaesthesia
Jacob et al^19^	RCT	Three groups‐ Group A: PRP injections; Group B: HA injections; Group C: Arthrocentesis alone	1‐, 3‐, 6‐month intervals	All groups saw pain reduction and mouth opening increase, with Group B leading in mouth opening. At the 6‐month follow‐up, post‐operative joint sounds were observed in three patients among the 16 in Group A, six patients out of 15 in Group B, and eight patients among the 16 in Group C.Differences in pain, mouth opening, and jaw movements across groups were not statistically significant
Manfredini et al.^20^	RCT	Six groups: (A) 1 ATS with SS, (B) 1 ATS with SS + 1 mL Triamcinolone, (C) 1 ATS with SS + 1 mL HA (600 kDa), (D) 1 ATS with SS + 1 mL HA (6000 kDa)—discontinued due to adverse effects, (E) 5 ATS + HA (600 kDa) sessions, F) 5 ATS + HA (600 kDa) sessions	End of treatment, 3 months	Improvements in all groups, with the most significant in Group E. Group D discontinued due to severe post‐injection pain
Møystad et al^21^	RCT	Group S: Sodium hyaluronate. Group C: Corticosteroid	2 weeks, 1 month, 6 months	Injections provided more relief to patients with joint pain only. Pain reduction was more in HA group than CS group. No significant difference in jaw movement or bone changes
Tang et al^22^	RCT	Group A: Five 1 mL injections of 1500–2500 kDa HA, 1 week apart. Group B: Five SS injections, 1 week apart. Control group: 20 healthy controls were also recruited	During each injection, 1 week after last injection	PAS levels in synovial fluid samples higher in osteoarthritis patients (Group A + control) than in healthy volunteers. Group A showed significant pain reduction and PAS concentration improvement than Group B. Adverse effects not mentioned
Toameh et al^23^	RCT	Three groups‐ 1. Arthrocentesis only (Control); 2. Arthrocentesis plus hyaluronic acid (HA); 3. Arthrocentesis plus platelet‐rich plasma (PRP)	1‐, 3‐, 6‐, and 9‐month follow‐ups	All groups had significant increases in maximum mouth opening (MMO) and masticatory efficiency, and decreases in pain intensity over the 9 months. PRP group showed the most improvement: MMO from 32.3 to 48.2 mm, masticatory efficiency from 3.9 to 5.1 on the VAS scale, and pain intensity decreased from 6.1 to 0.7 on the VAS scale. The HA group also improved significantly, and the control group showed smaller improvements

## AUTHOR CONTRIBUTIONS

P.K. data analysis. B.J.K conceptualization. M.M.M. writing and supervision. V.R. writing and supervision. M.C. revised and finalised the manuscript. G.M. writing, editing and supervision.

## FUNDING INFORMATION

This research received no external funding.

## CONFLICT OF INTEREST STATEMENT

The authors declare no conflict of interest.

### PEER REVIEW

The peer review history for this article is available at https://www.webofscience.com/api/gateway/wos/peer‐review/10.1111/joor.13840.

## Data Availability

The data will be available on reasonable request from the corresponding author.
